# Topochemical molten salt synthesis for functional perovskite compounds

**DOI:** 10.1039/c5sc03521j

**Published:** 2015-10-16

**Authors:** Lihong Li, Jinxia Deng, Jun Chen, Xianran Xing

**Affiliations:** a Department of Physical Chemistry , University of Science and Technology Beijing , Beijing 100083 , China . Email: Xing@ustb.edu.cn; b Key Laboratory of Green Printing , Institute of Chemistry , Chinese Academy of Sciences (ICCAS) , Beijing Engineering Research Center of Nanomaterials for Green Printing Technology , Beijing National Laboratory for Molecular Sciences (BNLMS) , Beijing 100190 , China

## Abstract

A topochemical molten salt synthesis (TMSS) method, which is rapid and a modification of the molten salt synthesis method (MSS), to facilitate crystal growth with improved phase-purity and particle homogeneity, is one of the strategic approaches aimed at a controllable morphology synthesis. The TMSS method is an environmentally friendly and mild way to prepare pure and controllable perovskites, which are famous functional materials, used as piezoelectrics, catalysts, ferroelectrics, multiferroics, and negative thermal expansion compounds, at a moderate reaction temperature in a short soaking time. This report reviews various TMSS reactions and their applications in fulfilling the demand for the tunable morphology of perovskite materials, such as one dimensional, two dimensional and three dimensional perovskites in molten salts, which mainly include: piezoelectrics, photocatalysts, negative thermal expansion matters and other functional perovskites. The double and layered perovskites obtained by TMSS methods are also discussed. We believe that a comprehensive understanding of the TMSS method for functional perovskites will definitely promote the development of a clean, efficient and tunable production process for advanced functional materials.

## Abstract 2

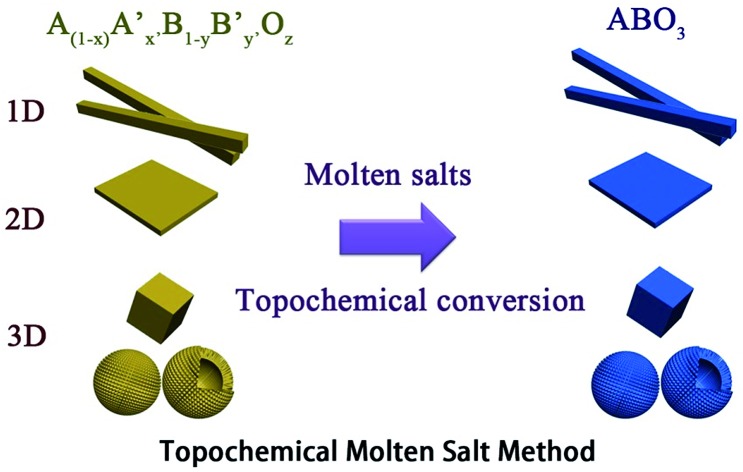
This report reviews various topochemical molten salt synthesis (TMSS) reactions and their applications in fulfilling the demand for the tunable morphology of perovskite materials.

## 1 Introduction

Due to the advancements in modern technology, the study of molten salt synthesis (MSS) has achieved considerable progress, and lots of salts have been found by chemists.^1^,^2^ MSS is one of the important issues especially for materials science. Salt melts have a long history as a solvent in research as well as in industry due to their low toxicity, low cost, low vapour pressure, abundant availability, high heat capacity, large electrochemical window, and high ionic conductivity.^3^ They have been used as reaction media for various organic and inorganic reactions, and also as the flux for crystal growth. Although, in previous studies, great success has been achieved in the study of the reaction mechanism of MSS and the nature of the salts, it still remains a challenge to extend the method to control the morphology of the target sample due to the difficult shape-control of the product in the high temperature molten salts.

The topochemical molten salt synthesis (TMSS) method is a kind of modification of the MSS method. It is one of the environmentally friendly and mild ways to prepare pure and morphologically-controllable samples at a moderate temperature in a short soaking time. The morphology of the products can inherit that of the major solid-state raw materials *via* TMSS, that is, the shape and size of the as-synthesized compounds can be controlled by an appropriate choice of raw materials, salts, sintering temperature and reaction time. The TMSS method has the advantages of combining the molten salt method and the topochemical method, associated with the use of localized solid-state compound transformations *via* the exchange, deletion, or insertion of individual atoms.^4^

Perovskites, a kind of famous functional materials, with potential as piezoelectrics, catalysts, multiferroics, solar cells and negative thermal expansion materials, have aroused much attention.^5^ However, a high synthesis temperature and long reaction time are usually needed to obtain them. Meanwhile, it is reported that they have unique shape-dependent properties, and many experimental efforts have been made to prepare hollow spheres, tubes, rods, and wires, as well as sheets and platelets. The TMSS gives us a useful strategy to modify the properties of the functional perovskite materials by being dependent on the nature of both of the salts and the raw materials. The TMSS’s low reaction temperature, short reaction time, tunable morphology allows a broad range of inorganic crystalline perovskites to be obtained. It is interesting to systematically discuss the TMSS method for the controllable morphology of functional perovskite compounds and the effect of the raw materials. The study on topochemical molten salt synthesizing for functionalized perovskite compounds is a new topic especially in the discipline of materials chemistry. It is timely to review the achievements and promote the development of TMSS applications for the future.^6^,^7^

In the present review, various TMSS reactions and their applications in fulfilling the demand for tunable morphology perovskite materials, such as one dimensional, two dimensional and three dimensional perovskites in molten salts, are summarized and discussed. It should be noted that the dimension/microstructure of the target sample is inherited from the different fusing of the raw materials. Meanwhile, the functional perovskites, which mainly include: piezoelectrics, photocatalysts, negative thermal expansion compounds and other functional perovskites, obtained from the TMSS method are expounded upon, with the purpose of providing a brief overview of the topochemical molten salt synthesis method and its influence on the energy efficiency, chemical composition or microstructure of the functional perovskite materials. In addition, the double and layered perovskites obtained by the TMSS methods and the perovskites synthesized by low temperature TMSS methods are also discussed. In the end, the possible further applications of the TMSS method for perovskites are predicted. We believe that a comprehensive understanding of the TMSS method for functional perovskites will definitely promote the development of a clean, efficient and tunable production process for advanced functional materials.

## 2 Morphology of perovskite compounds controlled by TMSS

### 2.1 The mechanism and method of the TMSS process

The synthesis of nanoscale structures with special morphologies has attracted extensive attention in the past two decades as a result of their novel size-dependent properties. Intense experimental efforts have been spent on preparing nanoparticles, nanowires, nanotubes, nanoplatelets and three dimensional particles of nanostructures.^8^ The use of different types of “fluxes”, including low melting metals and salts, has in fact been extensively explored for the synthesis of metallic and non-metallic materials in the form of either single crystals or polycrystalline powders.^9^–^22^ Compared to solid state reactions for which the rates are usually seriously limited by the slow diffusion of the reactants, the molten salt synthesis (MSS) method lowers the reaction temperature as it allows faster mass transfer transport in the liquid phase by means of convection and diffusion. As many salts by their nature dissolve in water, molten salt synthesis (MSS) has the advantage of easy isolation of the product.^1^ It is known that target morphology control is still a challenge for the MSS method.

**Fig. 1:**
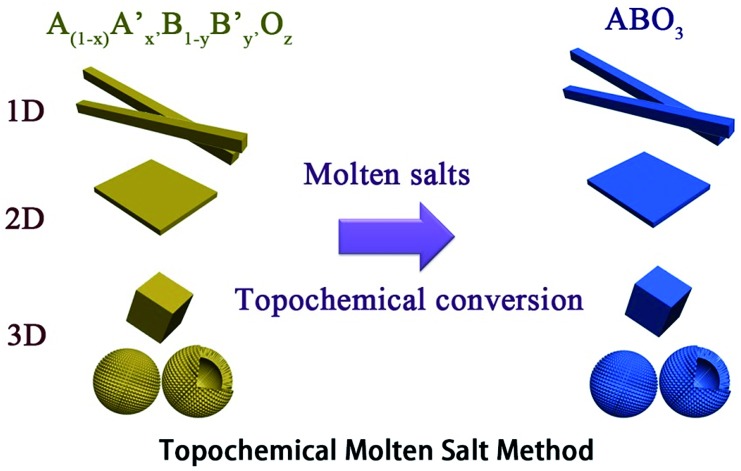
The schematic illustration of the topochemical molten salt method.

The TMSS route uses an inorganic salt heated above its melting temperature to serve as the solvent with partial solid-state raw materials. It is rapid, environmentally friendly and is similar to epitaxial growth, associated with using localized partial solid-state raw material transformations *via* the exchange, deletion, or insertion of individual atoms. In TMSS the morphology of the products can inherit that of the major refractory solid-state raw materials, that is the localized partial solid-state raw materials are used as self-templates. It should be pointed out that the partial solid-state raw materials or the precursors as templates should be refractory or micro-melting in the molten salts. It is interesting that, in some studies, pure samples could be obtained with controllable morphology in a few minutes. In contrast to the MSS, which needs a high solubility to obtain nanomaterials, the TMSS does not need all the precursors to be soluble and the reaction time is largely reduced. The involved schematic illustration of the topochemical molten salt method is summarized in Fig. 1.

The equilibrium is set up for the TMSS method by the following reaction:1
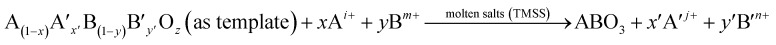
where the value of *x*, *x*′, *y* and *y*′ are all greater than or equal to 0 and less than or equal to 1, and *z* is a positive integer.

The performance of perovskite compounds in target applications is dramatically affected by the structure and morphology of the material. It is known that the shape of crystalline particles depends on their internal structure, which means that materials with a cubic structure will normally form isotropic particles.^23^ Regular-perovskite structured materials typically grow as equiaxed particles so it is difficult to synthesize anisotropic particles using conventional methods. The TMSS method is one of the strategic approaches aimed at controllable synthesis, which is associated with using localized solid-state raw materials. This type of topochemical method based on molten salt synthesis has been carried out to prepare various perovskite compounds with one dimension (1D), two dimensions (2D), and three dimensions (3D), such as, rodlike KNbO_3_/(Na,K)NbO_3_ using a Nb_2_O_5_ template, PbTiO_3_ using TiO_2_,^36^ platelike NaNbO_3_/KNbO_3_ using Bi_2.5_Na_3.5_Nb_5_O_18_,^44^,^49^,^61^ BaTiO_3_ using BaBi_4_Ti_4_O_15_,^38^ KNbO_3_ using K_4_Nb_6_O_17_, (Na,K)NbO_3_ using K_4_Nb_6_O_17_,^51^,^55^ and hollow sphere KNbO_3_ using Nb_2_O_5_.^57^ More details can be found in Table 1. Meanwhile, it is important to select the right molten salt system. It can be seen in Table 1, that the typical molten salts are metal halides and oxygenated chemicals. The reaction temperature should be higher than the melting point of the molten salts. To achieve a lower melting point, mixtures of two or more salts are usually used which provides a wider operating temperature range. It has to be pointed out that in some reactions, the molten salts not only play a role as the flux, but also join the reaction through the presence of certain cations or anions, such that Zn^2+^ from ZnCl_2_ can also be precipitated in the products and ZnEu_2_Ti_3_O_10_ was easily prepared by ion-exchanging K_2_Eu_2_Ti_3_O_10_ in molten ZnCl_2_ (melt point = 283 °C) at 300 °C.^60^ The equilibrium of it might be the following reaction:2
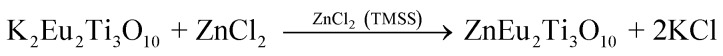



**Table 1:** A summary of different dimensional perovskites synthesized by the TMSS method and their templates

Dimensionality	Materials	Template	Molten salts	Reference
1D	NaNbO_3_	K_2_Nb_8_O_21_ nanowires	NaCl	24
		Nb_2_O_5_ nanorods	NaCl	25
	KNbO_3_	Nb_2_O_5_ nanorods	KCl	25
	LiNbO_3_	Nb_2_O_5_ nanowires	LiCl	26
	(K,Na)NbO_3_	Nb_2_O_5_ nanorods	KCl	25 and 27–30
	BaTiO_3_	TiO_2_ nanorods	NaCl–KCl (1 : 1 mol)	31
		H_2_Ti_3_O_7_ nanowires	Ba(OH)_2_	32–35
	PbTiO_3_	TiO_2_ rods	NaCl–KCl (1 : 1 mol)	36 and 37
2D	BaTiO_3_	BaBi_4_Ti_4_O_15_ platelets	NaCl–KCl (1 : 1 mol)	38
	SrTiO_3_	SrBi_4_Ti_4_O_15_ platelets	KCl	39
	CaTiO_3_	CaBi_4_Ti_4_O_15_ platelets	KCl	40
	Ba_1–*x*_Ca_*x*_TiO_3_	Bi_4_Ti_3_O_12_ platelets	NaCl–KCl (1 : 1 mol)	41
	Na_0.5_Bi_0.5_TiO_3_	Na_0.5_Bi_4.5_Ti_4_O_15_ platelets	NaCl–KCl (1 : 1 mol)	42 and 43
	NaNbO_3_	Bi_2.5_Na_3.5_Nb_5_O_18_ platelets	NaCl	44–47
		Na_3.5_Bi_2.5_Nb_5_O_18_ platelets	NaCl	7, 44 and 48
	KNbO_3_	K_4_Nb_6_O_17_ platelets	KCl	49 and 50
		Nb_2_O_5_ platelets	KCl	51
	(Na_0.5_K_0.5_)NbO_3_	K_4_Nb_6_O_17_ platelets	KCl	17
	AgSbO_3_	NaSbO_3_ nanoplates	AgNO_3_	52
	Ag_2_[Ca_1.5_M_3_O_10_] (M = Nb, Ta)	Li_2_[Ca_1.5_M_3_O_10_] (M = Nb, Ta)	AgNO_3_	53
	AgLaNb_2_O_7_	RbLaNb_2_O_7_	AgNO_3_	8 and 54
	AgA_2_Nb_3_O_10_ (A = Ca, Sr)	RbA_2_Nb_3_O_10_ (A = Ca, Sr)	AgNO_3_	46 and 55
	PbTiO_3_	PbBi_4_Ti_4_O_15_	KCl	7
	0.4(Na_1/2_Bi_1/2_)TiO_3_–0.6PbTiO_3_	PbBi_4_Ti_4_O_15_	NaCl	56
3D	KNbO_3_	Nb_2_O_5_ hollow nanospheres	KCl	57 and 58
	LaMnO_3_	Porous spherical Mn_2_O_3_	NaNO_3_–KNO_3_ (2 : 1 mol)	59
	ZnEu_2_Ti_3_O_10_	K_2_Eu_2_Ti_3_O_10_	ZnCl_2_	60
	AEu_2_Ti_2_NbO_10_ (A = Na, Li)	CsEu_2_Ti_2_NbO_10_	Alkali nitrates	60

### 2.2 One dimensional morphology

**Fig. 2:**
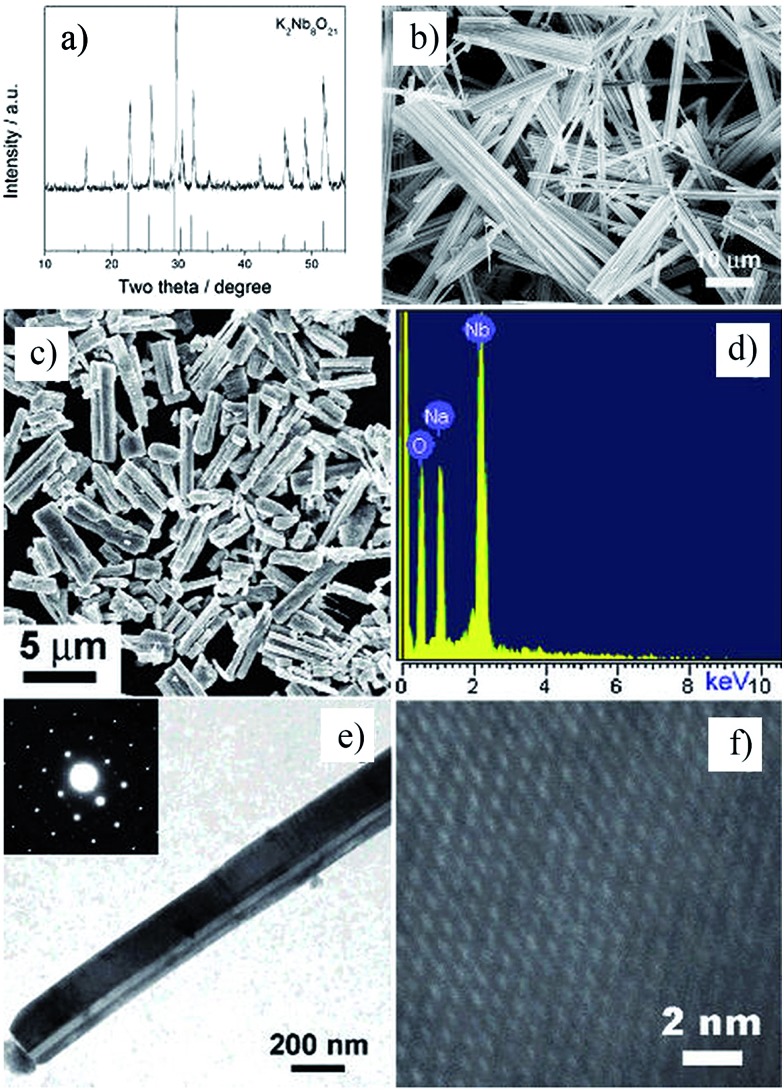
(a) XRD and (b) SEM images of the potassium niobate nanowires. The angles for the standard sample of potassium niobate are shown in the lower part of panel a. (c) SEM image of sodium niobate nanorods. (d) EDX spectrum showing the complete conversion from K niobate to Na niobate. (e) TEM image of an individual sodium niobate nanorod. Inset is the corresponding SAED pattern. (f) High-resolution TEM image of the same nanorod shown in panel e (ref. 32 Copyright 2007, American Chemical Society).

Nanorods, nanowires, nanotubes and other one dimensional materials have recently been investigated with increasing intensity as a result of their novel properties,^62^ which open up new paths for applications in several fields such as electronics, sensing, catalysis, energy harvesting and information storage. A number of articles on 1D nanostructures have been published,^8^,^26^ providing an outline of the research directions for the synthesis and applications of the 1D nanostructures. For example, subwavelength optical microscopy employing a tunable nanometric light source based on KNbO_3_ nanowires was developed by Yang and co-workers.^47^ Despite the attractive applications of niobates, there are only a few reports on the synthesis of niobate 1D nanostructures by employing the hydrothermal approach.^47^,^63^ Wang *et al.* have used the TMSS approach for the synthesis of single-crystal sodium and calcium niobate nanorods (Fig. 2).^24^ The synthesis of sodium and calcium niobate nanorods is a two-step process. First, K_2_Nb_8_O_21_ nanowires were prepared by calcination of Nb_2_O_5_ powders in molten KCl at 1000 °C for 3 h. Then, the mixture of the K_2_Nb_8_O_21_ nanowires and NaCl was heated in a tube furnace at 825 or 800 °C for 3 h and 1D sodium and calcium niobates were obtained based on this topochemical molten salt reaction between the K_2_Nb_8_O_21_ nanowires and the molten NaCl salt. The synthesized sodium niobate nanorods, with the same diameter of a few hundred nanowires as that of the precursor, and lengths of several micrometers, show a bundlelike morphology, which is characteristic of the starting K_2_Nb_8_O_21_ nanowires template. The phase of the obtained sodium niobate was determined to be orthorhombic NaNbO_3_ (JCPDS 33-1270), with lattice parameters of *a* = 0.5569, *b* = 1.5123, and *c* = 0.5505 nm.

### 2.3 Two dimensional morphology

**Fig. 3:**
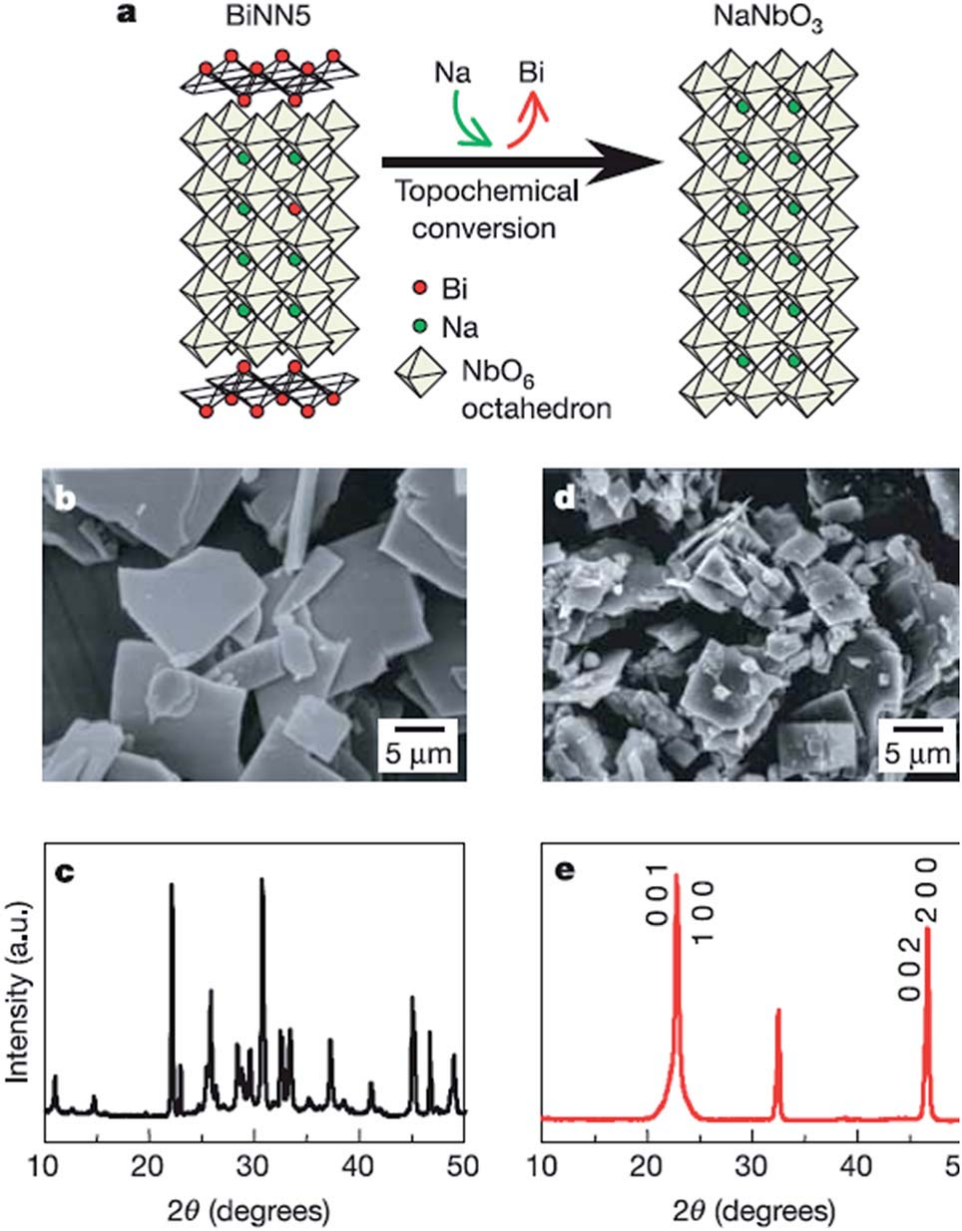
Schematic diagram of topochemical conversion from the bismuth layered structure BiNN5 particles to plate-like NaNbO_3_ particles. (a) Crystal structures of the plate-like BiNN5 and NaNbO_3_ particles. (b and c) An SEM image and the X-ray diffraction profile of the plate-like BiNN5 particles used as the precursor. (d and e) An SEM image and the X-ray diffraction profile of the plate-like NaNbO_3_ particles. The X-ray diffraction profile of the NaNbO_3_ particles is characterized by pseudo-tetragonal Miller indices. The BiNN5 particles are completely converted into the regular perovskite-structured NaNbO_3_ particles with a preserved plate-like shape (ref. 25 Copyright 2004 Nature Publishing Group.).

Two dimensional materials are materials in which the electron only has free movement in two dimensions, such as thin films, super lattices and quantum wells. The discovery of graphene largely promoted the development of 2D materials, and many researchers focus on the 2D materials used in solar cells, piezoelectric materials, *etc.*^64^ Recently, many efforts have been made to synthesize low dimensional perovskites, through techniques such as hydrothermal and molten salt synthesis (MSS), but the platelet perovskite particles are still not easily obtained due to the nature of regular-perovskite structured materials which typically grow as equiaxed particles. The TMSS method gives a route to obtain the 2D perovskites.^44^,^65^ For example, Saito *et al.* used the TMSS method to obtain plate-like NaNbO_3_ particles as templates for 001 oriented (K,Na)NbO_3_-based ceramics (Fig. 3). Firstly, a Bi_2.5_Na_3.5_Nb_5_O_18_ (BiNN5) platelet was synthesized at 1100 °C using molten NaCl salt as a flux. Then, using a TMSS reaction, a NaNbO_3_ platelet was synthesized from the BiNN5 and a complementary reactant, Na_2_CO_3_, in a NaCl flux at 950 °C. The by-product, Bi_2_O_3_, was removed. The synthesized NaNbO_3_ had the same morphology as BiNN5, a 0.5 mm thickness and 10–15 mm side length in a developed area, and consisted of a single-phase with the 001 plane of perovskite, identified by JCPDS powder diffraction file card no. 33-1270.^44^ The formation of the platelet NaNbO_3_ might be as follows:3
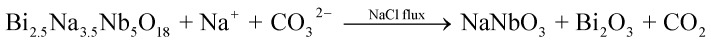



### 2.4 Three dimensional morphology

**Fig. 4:**
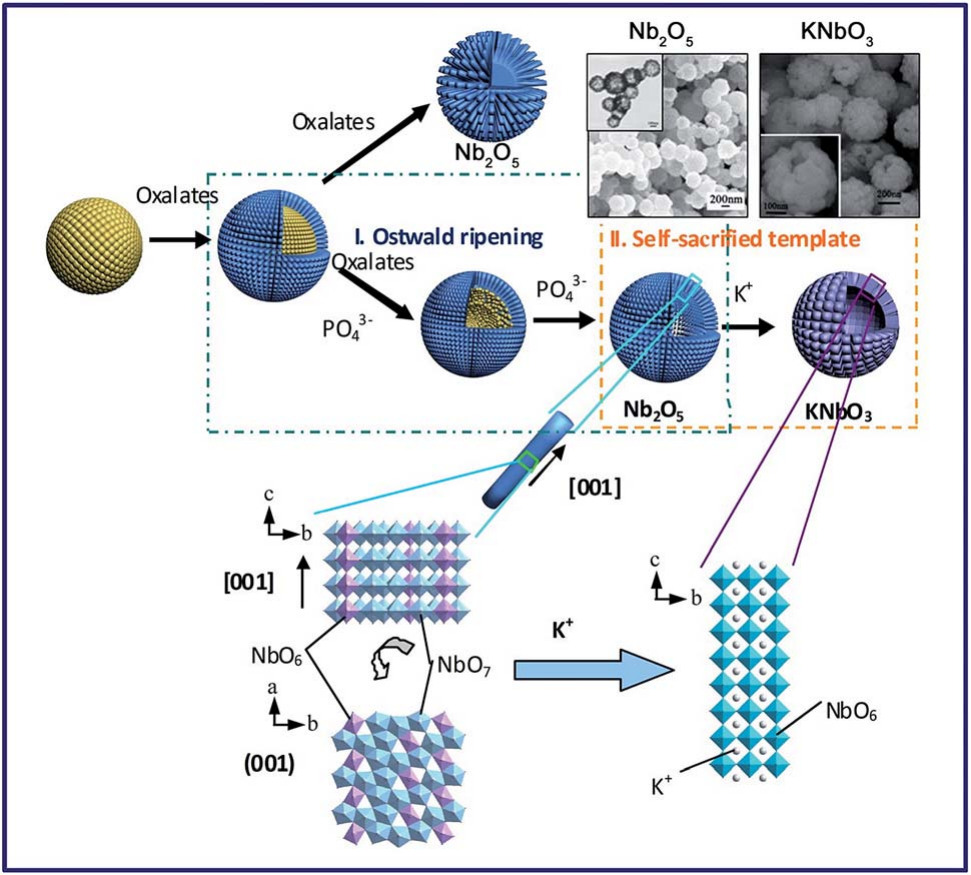
Formation processes and structure evolution of the KNbO_3_ hollow spheres and Nb_2_O_5_ solid spheres. The insets are the Nb_2_O_5_ hollow spheres and the KNbO_3_ hollow spheres (ref. 31 Copyright 2013, the Royal Society of Chemistry).

Materials with three dimensional morphologies, including composites consisting of one or more basic structural units in zero dimensional, one-dimensional, two-dimensional or porous materials, such as hollow spheres,^50^ nickel foam^66^*etc.*, have aroused much attention in the academic community and industry due to their unique microstructures and singular properties. The TMSS method has been used to obtain perovskites with 3D morphologies, for example, porous spherical and cubic LaMnO_3_ from porous Mn_2_O_3_ precursors in a mixture of NaNO_3_ and KNO_3_.^59^ There is another example, in which Xing *et al.* obtained KNbO_3_ hollow spheres from Nb_2_O_5_ hollow spheres *via* the TMSS method (Fig. 4).^57^ In the KCl molten salt, K^+^ needed to diffuse inside the T-Nb_2_O_5_ hollow spheres, and this process involved bond-breaking, rebonding, and the generation of new bonds. Viewed along the *c* axis of Nb_2_O_5_, the NbO_6_ and NbO_7_ units were corner-sharing, and in the perovskite KNbO_3_ crystal, the NbO_6_ octahedron units connected with shared corners along the *a*, *b*, and *c* axes. Therefore, although the small rods of the shell became cubelike, due to the high thermal stability of the Nb_2_O_5_ hollow nanospheres and the compatability of the structure of the Nb_2_O_5_ and KNbO_3_, the morphology of the hollow spheres could be kept and KNbO_3_ hollow nanospheres were obtained.

## 3 Various functional perovskites synthesized by TMSS

The ABO_3_ family with perovskite structure has aroused a broad interest since it was investigated in the 1970s.^67^ They have important applications in fields such as piezoelectrics, photocatalysis, ferroelectrics, multiferroics, and negative thermal expansion materials (NTE).^68^ The synthesis of these functional perovskites in the form of nano/micro crystalline powders and defined nanoscale architectures has been realized in different TMSS systems. Depending on the nature of the TMSS, low temperatures, short times, tunable morphologies, controllable stoichiometric ratios, and a broad range of inorganic crystalline structures of functional perovskites can be achieved.

### 3.1 Piezoelectric perovskites synthesized by TMSS

**Fig. 5:**
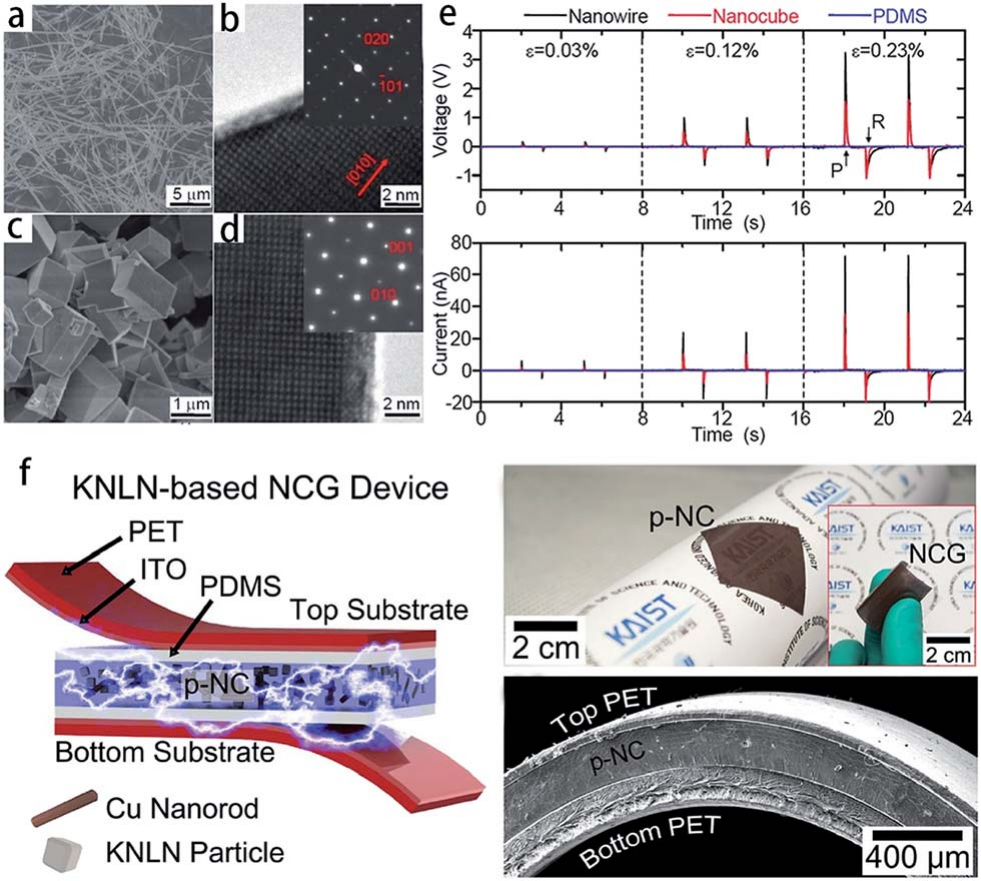
SEM and TEM images of NaNbO_3_ nanowires (a and b) and nanocubes (c and d). (e) Power generation of NaNbO_3_ nanowire-based (black lines) and nanocube-based (red lines) NGs at a given compressive strain. The top image is the open circuit voltage and below is the closed circuit current. PDMS itself (blue lines) does not show any signals. Here, *ε*, *P*, and *R* are for the strain value, press, and release, respectively. (f) Schematic of a flexible nanocomposite generator (NCG) device using 0.942 (K_0.480_Na_0.535_)NbO_3_–0.058LiNbO_3_ (KNLN) particles and Cu NRs. Photograph of the flexible p-NC layer attached to a rolled paper. The inset shows the final NCG device bent by fingers (ref. 71 Copyright 2014, 2011 American Chemical Society and ref. 72 Copyright 2014, WILEY-VCH Verlag GmbH & Co. KGaA, Weinheim).

**Fig. 6:**
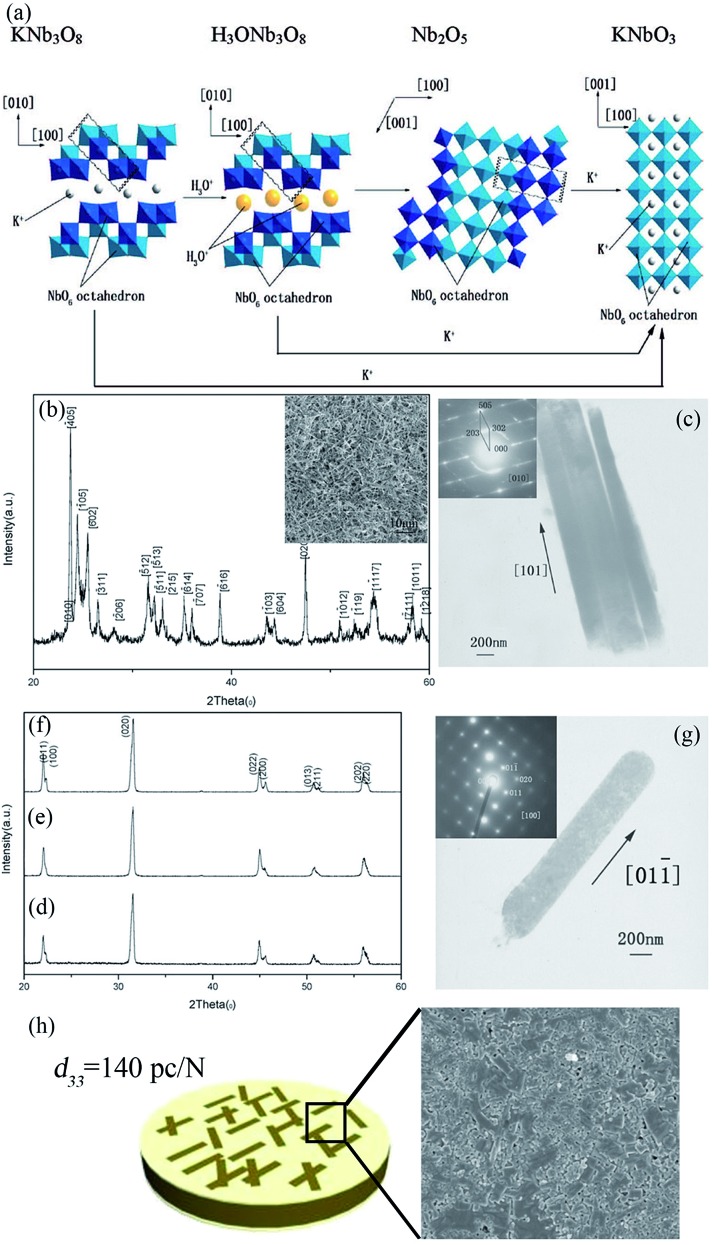
(a) Schematic illustration of the structural transformations of the Nb-containing species along with the mechanism of KNbO_3_ synthesis by TMSS treatment. (b) An XRD pattern of the Nb_2_O_5_ particles obtained from the rodlike H_3_ONb_3_O_8_ particles. The inset is an SEM micrograph of the Nb_2_O_5_ particles. (c) TEM image of the Nb_2_O_5_ rods with (inset) a typical SEAD pattern obtained from the rods. (d–f) XRD patterns of the KNbO_3_ particles obtained from different precursors KNb_3_O_8_, H_3_ONb_3_O_8_ and Nb_2_O_5_, respectively. (g) TEM image of an isolated KNbO_3_ rod obtained from the precursor Nb_2_O_5_ particles with (inset) its corresponding SEAD pattern. (h) The piezoelectric constant *d*_33_ of the (Na,K)NbO_3_ ceramic is 140 pC N^–1^, and the SEM image of the surface of the ceramic (ref. 33 Copyright 2009, American Chemical Society).

Piezoelectric materials can produce an electric field upon mechanical deformation, and form mechanical deformation *via* the effect of the electric field.^24^,^44^,^69^ The inherent mechanical electric coupling effect means piezoelectric materials have been widely used in nanometer generators,^70^ flexible nanocomposite generators (Fig. 5),^71^,^72^ sensors,^68^ actuators,^73^ and other electronic devices.^74^,^75^ Due to their significant piezoelectric properties the perovskite-type piezoelectric materials such as, Pb(Zr,Ti)O_3_ (PZT),^76^,^77^ Bi_0.5_Na_0.5_TiO_3_ (BNT),^69^,^78^ (K,Na)NbO_3_ (KNN),^44^,^72^ and BaTiO_3_ (BT)^72^ based materials, arouse widespread interest. The piezoelectric properties are largely affected by the morphology and structure of the materials (Fig. 5a–e),^45^,^71^ and the piezoelectric properties and morphology of the compounds can be tailored by the TMSS method. For example, the NaNbO_3_ platelet, synthesized by the TMSS method, is used as a reactive template for textured (K,Na)NbO_3_–LiTaO_3_(–LiSbO_3_) polycrystals synthesized by the reactive-templated grain growth (RTGG) method which exhibit a high piezoelectric constant *d*_33_ of 416 pC N^–1^.^44^ Rod-like ANbO_3_ (A = K, Na, (Na,K)) were fabricated by a TMSS method, shown in Fig. 6.^25^ The process is as follow: first, the precursor KNb_3_O_8_ with a rod structure was prepared under the conditions of a KCl molten salt environment at 800 °C for 3 h. Then, rod-like H_3_ONb_3_O_8_ and Nb_2_O_5_ were obtained from the rod-like KNb_3_O_8_ precursor. Finally, the rod-like ANbO_3_ (A = K, Na, (Na,K)) were achieved *via* the intermediate oxide Nb_2_O_5_. The rod-like structure of the final product can only be achieved when using the rod-like Nb_2_O_5_ precursor. The structural evolution investigated among protonic niobate, niobium oxide, and niobates, shows that the similar structure (three NbO_6_ octahedra connected with shared corners and edges along the [001] direction) is the key to maintaining the morphology of the precursor. The (Na,K)NbO_3_ ceramic sintered from the as-prepared rodlike particles under pressureless conditions in air performed with a high piezoelectricity (*d*_33_ = 140 pC N^–1^), which is much better than that of the ceramics obtained from cubic or spherical particles (*d*_33_ = 97 pC N^–1^).^25^

The reaction of the rod-like KNb_3_O_8_ might be as follows: first, the Nb_2_O_5_ and KCl were reacted to produce KNb_3_O_8_. Then an ion exchange of the K^+^ ion by the hydronium ion was observed according to the reaction:4
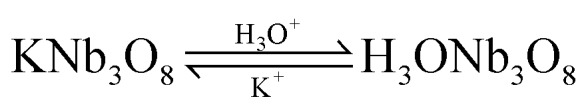



Afterward, the H_3_ONb_3_O_8_ was heated to remove H_2_O, which is depicted as follows:

5 H_3_ONb_3_O_8_ → HNb_3_O_8_ + H_2_O

6
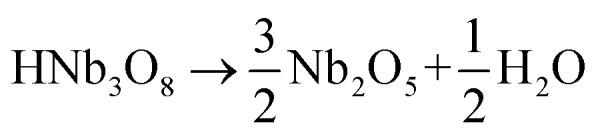



The reaction for the formation of the KNbO_3_ rods from the rodlike Nb_2_O_5_ particles is as follows:

7 Nb_2_O_5_ + K_2_CO_3_ → Nb_2_O_5_ + K_2_O + CO_2_ → 2KNbO_3_ + CO_2_

### 3.2 Photocatalysis perovskites synthesized by TMSS

**Fig. 7:**
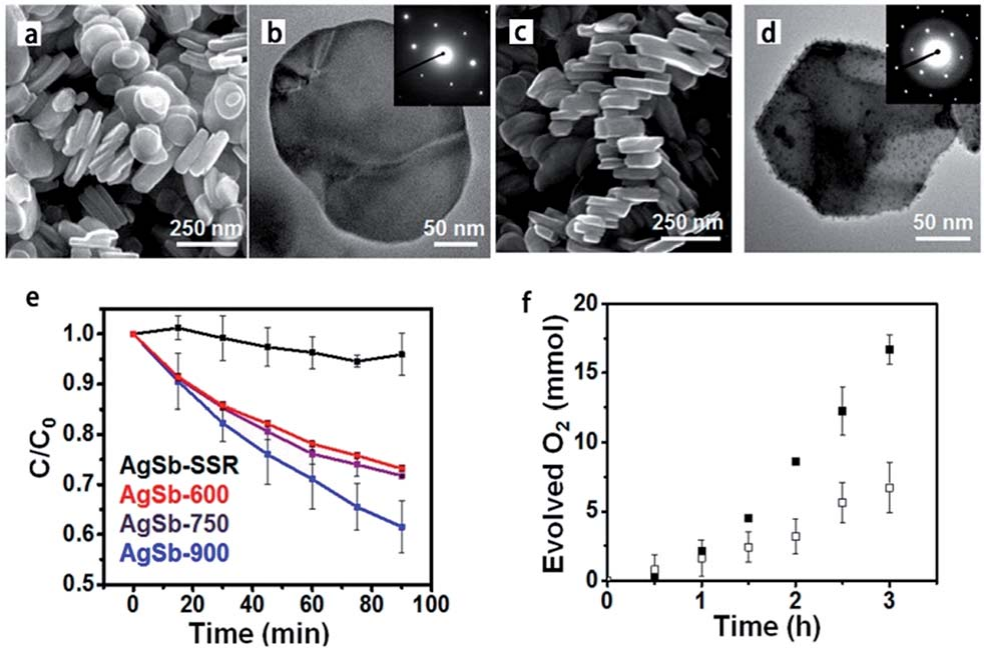
(a) SEM and (b) TEM images of the NaSbO_3_ product obtained from an aerosol-assisted synthesis. (c) SEM and (d) TEM images of the AgSbO_3_, single-crystalline NaSbO_3_ plates in molten AgNO_3_ were used as the template. (e) Rhodamine B photocatalytic degradation under *λ* = 400–500 nm illumination. (f) Oxygen evolution in the presence of 0.1 M AgNO_3_ (ref. 55 Copyright 2015, American Chemical Society).

Current synthetic challenges for the crystal growth of complex oxides can be addressed by utilizing TMSS methods, which have made a significant impact in research involving solar energy conversion.^30^,^52^,^79^ The improved phase-purity and particle homogeneity is in favour of enhancing photocatalytic properties. Early investigations into the photocatalytic activities of metal oxides utilized only high-temperature ‘grind and heat’ solid state syntheses to obtain the desired products.^80^ Starting with the research of the Maggard group, the MSS of metal-oxide photocatalysts has been used increasingly to understand the impact of the particle sizes, morphologies, and the surface.^81^,^82^ The TMSS preparation of perovskites has been of increasing importance in a growing number of studies probing photocatalytic mechanisms and the surface reactivities of photocatalysts, such as AgSbO_3_,^52^ LaMnO_3_,^59^ KNbO_3_ (ref. 57) and AgTaO_3_, AFeO_3_ (A = Bi, La, Ln), and Bi(Mg_3/8_Fe_2/8_Ti_3/8_)O_3_.^83^ For example, AgSbO_3_ visible-light photocatalysts were synthesized from NaSbO_3_ nanoplates, which were prepared by salt-assisted aerosol combustion, *via* the TMSS method (Fig. 7).^52^ It was revealed that the surface chemistry and particle morphology influenced the photocatalytic activity. Visible-light-induced photodegradation of RhB was selected as the model reaction to evaluate the photocatalytic properties of the different AgSbO_3_ samples. AgSbO_3_ prepared *via* the TMSS route exhibited a greater photoactivity for the photodegradation of RhB in comparison to the AgSbO_3_ synthesized from the other method (Fig. 7e). It can be predicted that further modifications of the TMSS methods for tuning particle sizes, morphologies, and specific surface areas could be used to obtain other nano perovskites with highly desired optical and photocatalytic properties in future.

### 3.3 Negative thermal expansion (NTE) perovskites synthesized by TMSS

The thermal expansion compatibility of different components is one of the key problems for modern devices, such as thin films, multilayer chip capacitors (MLCCs), solid oxide fuel cells, thermoelectric materials, and high temperature piezoelectrics. Thermal stress could give rise to device failure due to an undesirable mismatch of the coefficients of thermal expansion (CTE). The design of highly reliable devices should pay enough attention to the control of thermal expansion, which is extremely difficult, but an important topic. The size effect brings about unexpected phenomena upon thermal expansion. With decreasing particle sizes, NTE can be produced in those compounds which have normal thermal expansion in the bulk state, such as magnetic nanoparticles.^84^ Moreover, giant NTE of ferroelectrics is lost and transformed to a positive thermal expansion, as particle size decreases. The development of TMSS can reveal the nature of NTE perovskites as the particle size, stoichiometric ratio and dispersity can be controlled *via* this chemical method. Perovskite type NTE materials were synthesized by the TMSS method, for example PbTiO_3_ (ref. 36) and PbTiO_3_-based materials.^56^ It should be mentioned that the effect of size on thermal expansion is just beginning to be studied. More interesting results will appear, and the research in this field is significant for the development of devices, as the nano/micro materials for devices become important.^84^

### 3.4 Other functional perovskites synthesized by TMSS

Other functional perovskites, such as multiferroic materials, catalysts *etc.* are also prepared *via* the TMSS method. For perovskite-type multiferroic materials, they have potential applications for new types of electronic devices, such as ferroelectrics, multiple-state memories and new data-storage media.^85^–^87^ The synthesis method used to obtain the desired nanostructures is crucial for exploiting nanoscale electric, magnetic, and thermal properties. For example, for the multiferroic BiFeO_3_-based material, the limited available information relating to the size dependence of the physical properties is mainly due to the difficulty in preparing the pure BiFeO_3_-based material.^88^ During the synthesis of BiFeO_3_, the kinetics of the phase formation in the Bi_2_O_3_–Fe_2_O_3_ system can easily lead to the appearance of impurity phases, such as Bi_25_FeO_40_ and Bi_2_Fe_4_O_9_. The TMSS is an appropriate and rapid method for such materials. Not only high purity samples, but also samples with controllably sized particles can be obtained.^86^,^89^ Perovskite-type catalysts, are promising automotive exhaust catalysts^90^ for the catalytic removal of VOCs^59^*etc.* due to their surface redox properties, high bulk oxygen mobility and good thermal stability. The morphology and surface area have a great impact on the performance of catalysts. For example, the porous spherical and cubic LaMnO_3_ with a high activity for the catalytic removal of toluene was produced in a morphologically controlled synthesis *via* the TMSS method from the porous spherical M_2_O_3_.^59^

## 4 Double and layered perovskites synthesized by TMSS

Double perovskite oxides with the general formula AA_0_BB_0_O_6_ (where A and A_0_ are rare earth or alkaline earth metals, and B and B_0_ are d-block transition metals) display a wide variety of interesting physical properties that vary with changes in their composition. Considerable research is being carried out to explore new double perovskite materials, to understand the origin of their properties (*e.g.* magnetodielectric, magnetoresistance, and magneto-capacitance), to improve their properties, and to adapt the materials to produce technology for each application. The nanostructure of double perovskites can significantly enhance their properties. To obtain nanostructures, such as, La_2_CoMnO_6_, La_2_NiMnO_6_, Ca_2_Fe_0.8_Co_0.2_MoO_6_, the TMSS method has been applied due to the tunable morphology and the controllable stoichiometric ratio.^89^,^91^,^92^

**Fig. 8:**
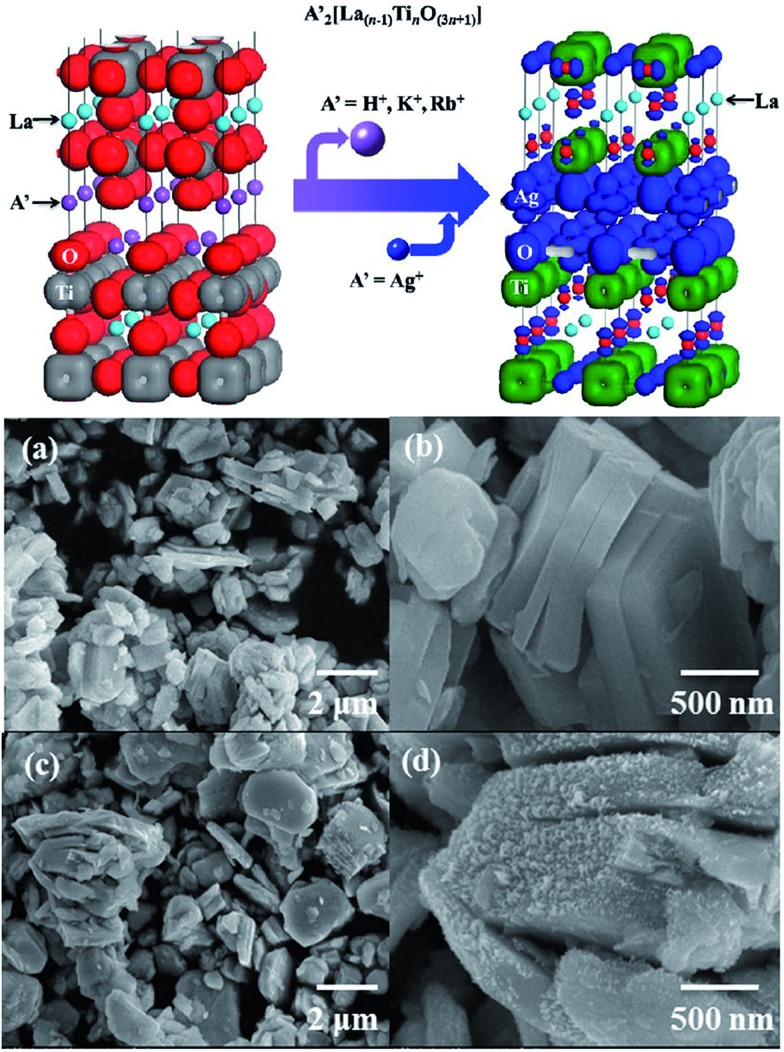
(Top) Electron density plot of A′_2_[La_(*n*–1)_Ti_*n*_O_(3*n*+1)_] and Ag_2_La_2_Ti_3_O_10_. (a and b) FESEM images of solid-state prepared Rb_2_La_2_Ti_3_O_10_. (c and d) Silver-exchanged Ag_2_La_2_Ti_3_O_10_ in AgNO_3_ flux (ref. 30 Copyright 2013, American Chemical Society).

Members of the Dion–Jacobson family of layered perovskites,^93^–^95^ A[A′_*n*–1_B_*n*_O_3*n*+1_] (A = alkali, A′ = alkaline earth or rare earth, B = transition metal), have an equal number of A(A′) and B cations, so they are ideal precursors to nondefective, three dimensional (3D) perovskites of the general formula AA′_*n*–1_BnO_3*n*_.^60^ For example, ZnEu_2_Ti_3_O_10_ was prepared by ion-exchanging K_2_Eu_2_Ti_3_O_10_ in molten ZnCl_2_. AEu_2_Ti_2_NbO_10_ (A = Na, Li) compounds were prepared from CsEu_2_Ti_2_NbO_10_ in the molten alkali nitrates. The conversion of a Dion–Jacobson layered perovskite A[A′_*n*–1_B_*n*_O_3*n*+1_] to a 3D perovskite AA′_*n*–1_B_*n*_O_3*n*_ requires that either the A, A′, or B ion be reducible.^60^ Furthermore, it is well known that the photocatalytic efficiency of a given semiconductor photocatalyst depends on three physical processes, including the light absorption, the transport of the charge carriers and the recombination of the photogenerated electron–hole pairs. The electronic band structure plays a critical role in the above processes. Furthermore, it was found that the electronic band structure of layered perovskites is able to be engineered.^96^ Boltersdorf *et al.* systematically reported that Ag_2_La_2_Ti_3_O_10_, and AgLaNb_2_O_7_, AgA_2_Nb_3_O_10_ (A = Ca, Sr) were prepared by the TMSS method using solid-state prepared Rb_2_La_2_Ti_3_O_10_ in AgNO_3_ salts (Fig. 8), and it was found that the silver-exchanged RbA_2_Nb_3_O_10_ layered structures exhibited the highest photocatalytic hydrogen formation rates under ultraviolet and visible irradiation (∼13 616 μmol H_2_ g^–1^ h^–1^).^55^

## 5 Conclusions and outlook

Synthesizing materials with the desired morphology and phase purity in a reproducible and environmentally friendly manner is arousing considerable attention in materials science all over the world. After two decades of studies on the topic of TMSS, there has been great progress in aspects of new TMSS processes for different perovskites. The new interdisciplinary field of TMSS for perovskites has been reviewed. The mechanism of the TMSS method is also presented. The properties, such as, piezoelectric, photocatalytic, negative thermal expansion *etc.*, and the different morphologies of perovskite compounds could be tailored by the TMSS method. Meanwhile, the double and layered perovskites can also be obtained by TMSS methods with a tunable morphology and a controllable stoichiometric ratio.

For future applications of TMSS in perovskites, there still exist some problems which have to be considered. For example, the solubility of different precursors and the understanding of the growth mechanisms of perovskites are still not fully developed; improved understanding of the chemical and physical properties and the crystal structures of the materials is called for. Further study of the TMSS method for tuning particle morphologies, size and crystallinity can be used to obtain other target morphologies of perovskites that are doped or have highly desirable piezoelectric, photocatalytic, negative thermal expansion properties *etc.* In addition, not limited to the preparation of perovskites, the TMSS method can be extended to obtain other kinds of functional materials, for example, a high voltage layered Li_1.2_Ni_0.16_Co_0.08_Mn_0.56_O_2_ cathode material with a hollow spherical structure has been synthesized by the TMSS method in a NaCl flux from MnO_2_ hollow spheres.^97^ The development of lower melting temperature molten salts (ionic liquids) for the TMSS method is anticipated to guide us to new synthetic protocols.

## References

[cit1] Liu X., Fechler N., Antonietti M. (2013). Chem. Soc. Rev..

[cit2] Xiao W., Wang D. (2014). Chem. Soc. Rev..

[cit3] Plechkova N. V., Seddon K. R. (2008). Chem. Soc. Rev..

[cit4] Schaak R. E., Mallouk T. E. (2002). Chem. Mater..

[cit5] Maeno Y., Hashimoto H., Yoshida K., Nishizaki S., Fujita T., Bednorz J. G., Lichtenberg F. (1994). Nature.

[cit6] Pan Z., Wang Q., Chen J., Liu C., Fan L., Liu L., Fang L., Xing X. (2015). J. Am. Ceram. Soc..

[cit7] Poterala S. F., Chang Y., Clark T., Meyer R. J., Messing G. L. (2010). Chem. Mater..

[cit8] Xia Y., Yang P., Sun Y., Wu Y., Mayers B., Gates B., Yin Y., Kim F., Yan H. (2003). Adv. Mater..

[cit9] Kanatzidis M. G., Pöttgen R., Jeitschko W. (2005). Angew. Chem., Int. Ed..

[cit10] An X., Wang Y., Deng J., Chen J., Xing X. (2014). Mater. Res. Bull..

[cit11] An X., Wang Y., Deng J., Chen J., Yu R., Xing X. (2014). Inorg. Chem. Commun..

[cit12] Cai Z., Xing X., Yu R., Liu G., Xing Q. (2006). J. Alloys Compd..

[cit13] Chen J., Zhang C., Xing X. (2008). Mater. Lett..

[cit14] Deng J., Chen J., Yu R., Liu G., Xing X. (2009). Int. J. Mater. Res..

[cit15] Deng J. X., Li L. H., Chen J., Yu R. B., Xing X. R. (2012). Inorg. Chem. Commun..

[cit16] Hu L., Chen J., Fan L., Deng J., Yu R., Xing X. (2014). J. Am. Ceram. Soc..

[cit17] Li L. H., Chen J., Deng J. X., Yu R. B., Qiao L. J., Liu G. R., Xing X. R. (2008). Eur. J. Inorg. Chem..

[cit18] Li L. H., Deng J. X., Chen J., Yu R. B., Qiao L. J., Xing X. R. (2009). J. Alloys Compd..

[cit19] Li L. H., Xing X. R., Chen J., Cai Z. Y., Deng J. X., Sun C., Liu G. R. (2006). Chin. J. Inorg. Chem..

[cit20] Xing X., Dai S., Zhu Z., Tanaka T. (2002). Thermochim. Acta.

[cit21] Xing X., Zhang C., Qiao L., Liu G., Meng J. (2006). J. Am. Ceram. Soc..

[cit22] Xing X., Zhu Z., Dai S., Tanaka T. (2001). Thermochim. Acta.

[cit23] Pribošič I., Makovec D., Drofenik M. (2005). Chem. Mater..

[cit24] Xu C.-Y., Zhen L., Yang R., Wang Z. L. (2007). J. Am. Chem. Soc..

[cit25] Li L. H., Deng J. X., Chen J., Sun X. Y., Yu R. B., Liu G. R., Xing X. R. (2009). Chem. Mater..

[cit26] Santulli A. C., Zhou H., Berweger S., Raschke M. B., Sutter E., Wong S. S. (2010). CrystEngComm.

[cit27] Cheng L.-Q., Wang K., Li J.-F. (2013). Chem. Commun..

[cit28] Cheng L.-Q., Wang K., Yu Q., Li J.-F. (2014). J. Phys. Chem. C.

[cit29] Cheng L.-Q., Wang K., Li J.-F., Liu Y., Li J. (2014). J. Phys. Chem. C.

[cit30] Boltersdorf J., King N., Maggard P. A. (2015). CrystEngComm.

[cit31] Huang K.-C., Huang T.-C., Hsieh W.-F. (2009). Inorg. Chem..

[cit32] Lee D. K., Cho I.-S., Lee S., Kim D. H., Shim H.-W., Kim D.-W., Hong K. S. (2010). Eur. J. Inorg. Chem..

[cit33] Dupont J., de Souza R. F., Suarez P. A. Z. (2002). Chem. Rev..

[cit34] Cohen R. E. (1992). Nature.

[cit35] Cheong S.-W., Mostovoy M. (2007). Nat. Mater..

[cit36] Cai Z., Xing X., Yu R., Sun X., Liu G. (2007). Inorg. Chem..

[cit37] Lee M. M., Teuscher J., Miyasaka T., Murakami T. N., Snaith H. J. (2012). Science.

[cit38] Liu D., Yan Y., Zhou H. (2007). J. Am. Ceram. Soc..

[cit39] Saito Y., Takao H. (2006). Jpn. J. Appl. Phys..

[cit40] Saito Y., Takao H., Wada K. (2008). Ceram. Int..

[cit41] Yan X., Gao F., Liu Z. (2014). Ceram. Int..

[cit42] Zhao W., Zhou H., Yan Y., Liu D. (2008). J. Am. Ceram. Soc..

[cit43] Hussain A., Rahman J. U., Ahmed F., Kim J.-S., Kim M.-H., Song T.-K., Kim W.-J. (2015). J. Eur. Ceram. Soc..

[cit44] Saito Y., Takao H., Tani T., Nonoyama T., Takatori K., Homma T., Nagaya T., Nakamura M. (2004). Nature.

[cit45] Zeng W., Tao X.-M., Chen S., Shang S., Chan H. L. W., Choy S. H. (2013). Energy Environ. Sci..

[cit46] Yan Y., Liu D., Zhao W., Zhou H., Fang H. (2007). J. Am. Ceram. Soc..

[cit47] Nakayama Y., Pauzauskie P. J., Radenovic A., Onorato R. M., Saykally R. J., Liphardt J., Yang P. (2007). Nature.

[cit48] Wang Z. L., Song J. H. (2006). Science.

[cit49] Saito Y., Takao H. (2007). J. Eur. Ceram. Soc..

[cit50] Caruso F., Caruso R. A., Möhwald H. (1998). Science.

[cit51] Li L. H., Deng J. X., Yu R. B., Chen J., Wang X. W., Xing X. R. (2010). Inorg. Chem..

[cit52] Chen D. P., Bowers W., Skrabalak S. E. (2015). Chem. Mater..

[cit53] Bhuvanesh N. S. P., Woodward P. M. (2002). J. Am. Chem. Soc..

[cit54] Arney D., Maggard P. A. (2012). ACS Catal..

[cit55] Boltersdorf J., Maggard P. A. (2013). ACS Catal..

[cit56] Poterala S. F., Meyer J. R. J., Messing G. L. (2011). J. Am. Ceram. Soc..

[cit57] Li L. H., Deng J. X., Yu R. B., Chen J., Wang Z., Xing X. R. (2013). J. Mater. Chem. A.

[cit58] Gao P. X., Song J., Liu J., Wang Z. L. (2007). Adv. Mater..

[cit59] Wang Y., Xie S., Deng J., Deng S., Wang H., Yan H., Dai H. (2014). ACS Appl. Mater. Interfaces.

[cit60] Schaak R. E., Mallouk T. E. (2000). J. Am. Chem. Soc..

[cit61] Gao T., Liao J., Wang J., Qiu Y., Yang Q., Zhang M., Zhao Y., Qin L., Xue H., Xiong Z., Chen L., Wang Q.-M. (2015). J. Phys. Chem. A.

[cit62] Einarsrud M.-A., Grande T. (2014). Chem. Soc. Rev..

[cit63] Magrez A., Vasco E., Seo J. W., Dieker C., Setter N., Forró L. (2006). J. Phys. Chem. B.

[cit64] Coleman J. N., Lotya M., O'Neill A., Bergin S. D., King P. J., Khan U., Young K., Gaucher A., De S., Smith R. J., Shvets I. V., Arora S. K., Stanton G., Kim H.-Y., Lee K., Kim G. T., Duesberg G. S., Hallam T., Boland J. J., Wang J. J., Donegan J. F., Grunlan J. C., Moriarty G., Shmeliov A., Nicholls R. J., Perkins J. M., Grieveson E. M., Theuwissen K., McComb D. W., Nellist P. D., Nicolosi V. (2011). Science.

[cit65] Lv D., Zuo R., Su S. (2012). J. Am. Ceram. Soc..

[cit66] Schaedler T. A., Jacobsen A. J., Torrents A., Sorensen A. E., Lian J., Greer J. R., Valdevit L., Carter W. B. (2011). Science.

[cit67] Libby W. F. (1971). Science.

[cit68] Zhu J., Li H., Zhong L., Xiao P., Xu X., Yang X., Zhao Z., Li J. (2014). ACS Catal..

[cit69] Zhang J., Pan Z., Guo F.-F., Liu W.-C., Ning H., Chen Y. B., Lu M.-H., Yang B., Chen J., Zhang S.-T., Xing X., Rödel J., Cao W., Chen Y.-F. (2015). Nat. Commun..

[cit70] Wang Z. L., Song J. (2006). Science.

[cit71] Jung J. H., Lee M., Hong J.-I., Ding Y., Chen C.-Y., Chou L.-J., Wang Z. L. (2011). ACS Nano.

[cit72] Jeong C. K., Park K.-I., Ryu J., Hwang G.-T., Lee K. J. (2014). Adv. Funct. Mater..

[cit73] Muralt P., Polcawich R. G., Trolier-McKinstry S. (2009). MRS Bull..

[cit74] Wu W., Wen X., Wang Z. L. (2013). Science.

[cit75] Wu W., Wang L., Li Y., Zhang F., Lin L., Niu S., Chenet D., Zhang X., Hao Y., Heinz T. F., Hone J., Wang Z. L. (2014). Nature.

[cit76] Kang H., Chen J., Liu L., Hu C., Fang L., Xing X. (2013). Inorg. Chem. Commun..

[cit77] Park K.-I., Son J. H., Hwang G.-T., Jeong C. K., Ryu J., Koo M., Choi I., Lee S. H., Byun M., Wang Z. L., Lee K. J. (2014). Adv. Mater..

[cit78] Zhang S.-T., Kounga A. B., Aulbach E., Ehrenberg H., Roedel J. (2007). Appl. Phys. Lett..

[cit79] An X., Deng J., Chen J., Xing X. (2013). Mater. Res. Bull..

[cit80] Domen K., Yoshimura J., Sekine T., Tanaka A., Onishi T. (1990). Catal. Lett..

[cit81] Arney D., Porter B., Greve B., Maggard P. A. (2008). J. Photochem. Photobiol., A.

[cit82] Arney D., Hardy C., Greve B., Maggard P. A. (2010). J. Photochem. Photobiol., A.

[cit83] Zhang W., Chen J., An X., Wang Q., Fan L., Wang F., Deng J., Yu R., Xing X. (2014). Dalton Trans..

[cit84] Chen J., Hu L., Deng J., Xing X. (2015). Chem. Soc. Rev..

[cit85] Eerenstein W., Mathur N. D., Scott J. F. (2006). Nature.

[cit86] Wang J., Neaton J. B., Zheng H., Nagarajan V., Ogale S. B., Liu B., Viehland D., Vaithyanathan V., Schlom D. G., Waghmare U. V., Spaldin N. A., Rabe K. M., Wuttig M., Ramesh R. (2003). Science.

[cit87] Grinberg I., West D. V., Torres M., Gou G., Stein D. M., Wu L., Chen G., Gallo E. M., Akbashev A. R., Davies P. K., Spanier J. E., Rappe A. M. (2013). Nature.

[cit88] Fiebig M., Lottermoser T., Frohlich D., Goltsev A. V., Pisarev R. V. (2002). Nature.

[cit89] Chen J., Xing X., Watson A., Wang W., Yu R., Deng J., Yan L., Sun C., Chen X. (2007). Chem. Mater..

[cit90] Xu G., Bai H., Huang X., He W., Li L., Shen G., Han G. (2015). J. Phys. Chem. A.

[cit91] Mao Y., Parsons J., McCloy J. S. (2013). Nanoscale.

[cit92] Chen J., Yu R., Li L., Sun C., Zhang T., Chen H., Xing X. (2008). Eur. J. Inorg. Chem..

[cit93] Dion M., Ganne M., Tournoux M. (1981). Mater. Res. Bull..

[cit94] Treacy M. M. J., Rice S. B., Jacobson A. J., Lewandowski J. T. (1990). Chem. Mater..

[cit95] Jacobson A. J., Johnson J. W., Lewandowski J. T. (1985). Inorg. Chem..

[cit96] Meng F., Hong Z., Arndt J., Li M., Zhi M., Yang F., Wu N. (2012). Nano Res..

[cit97] He X., Wang J., Kloepsch R., Krueger S., Jia H., Liu H., Vortmann B., Li J. (2014). Nano Res..

